# Supplementary material for: TGF-Beta Induces Activin A Production in Dermal Fibroblasts Derived from Patients with Fibrodysplasia Ossificans Progressiva
Source: Int J Mol Sci. 2023 Jan 24;24(3):2299. doi: 10.3390/ijms24032299 (PMC9916423; doi:10.3390/ijms24032299)
Supplement: Supplementary file 1 [file ijms-24-02299-s001.zip › All article figures v2.2.pptx]

## Slide 1
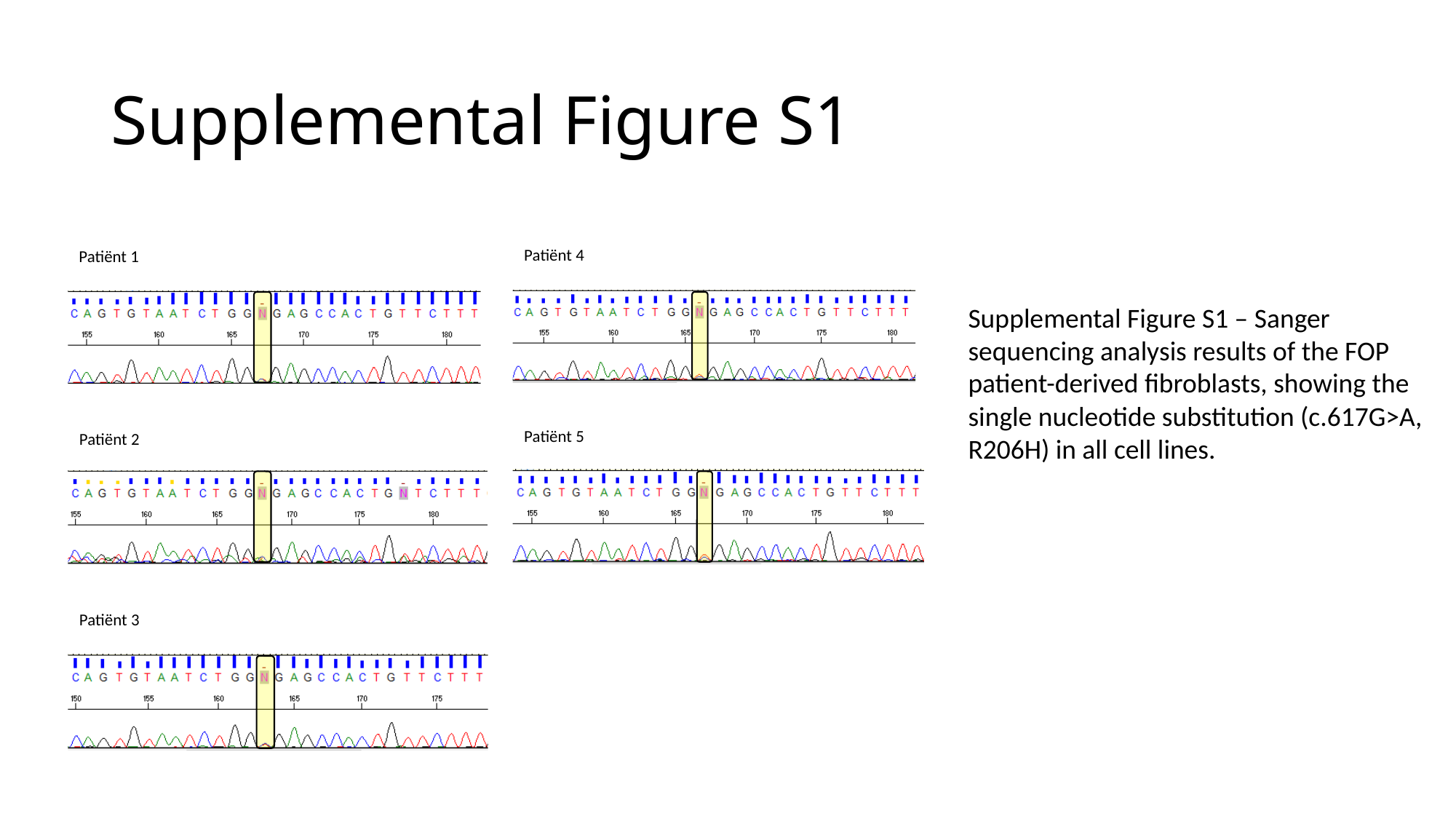

# Supplemental Figure S1
Patiënt 4
Patiënt 1
Supplemental Figure S1 – Sanger sequencing analysis results of the FOP patient-derived fibroblasts, showing the single nucleotide substitution (c.617G>A, R206H) in all cell lines.
Patiënt 5
Patiënt 2
Patiënt 3

## Slide 2
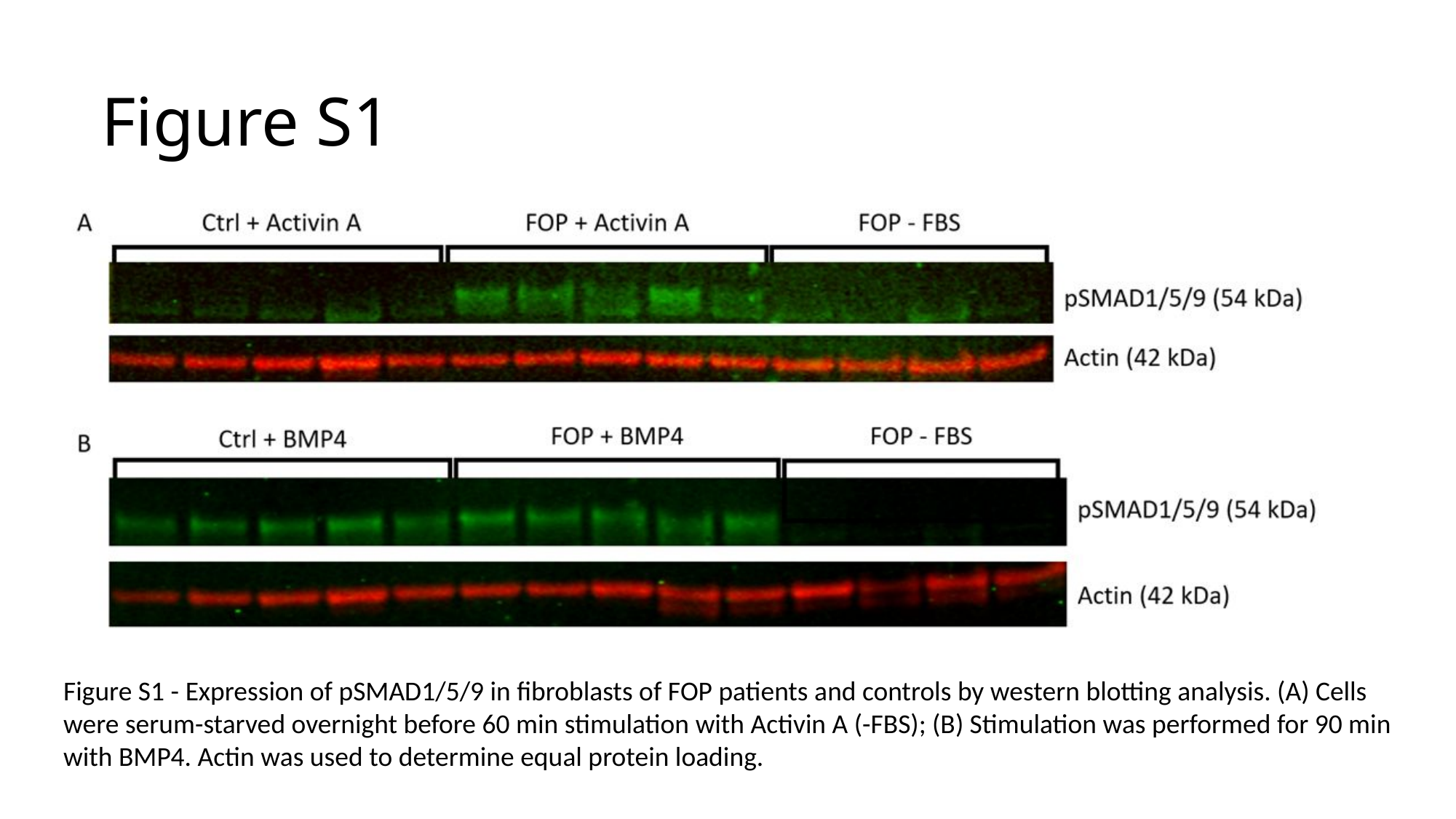

# Figure S1
Figure S1 - Expression of pSMAD1/5/9 in fibroblasts of FOP patients and controls by western blotting analysis. (A) Cells were serum-starved overnight before 60 min stimulation with Activin A (-FBS); (B) Stimulation was performed for 90 min with BMP4. Actin was used to determine equal protein loading.

## Slide 3
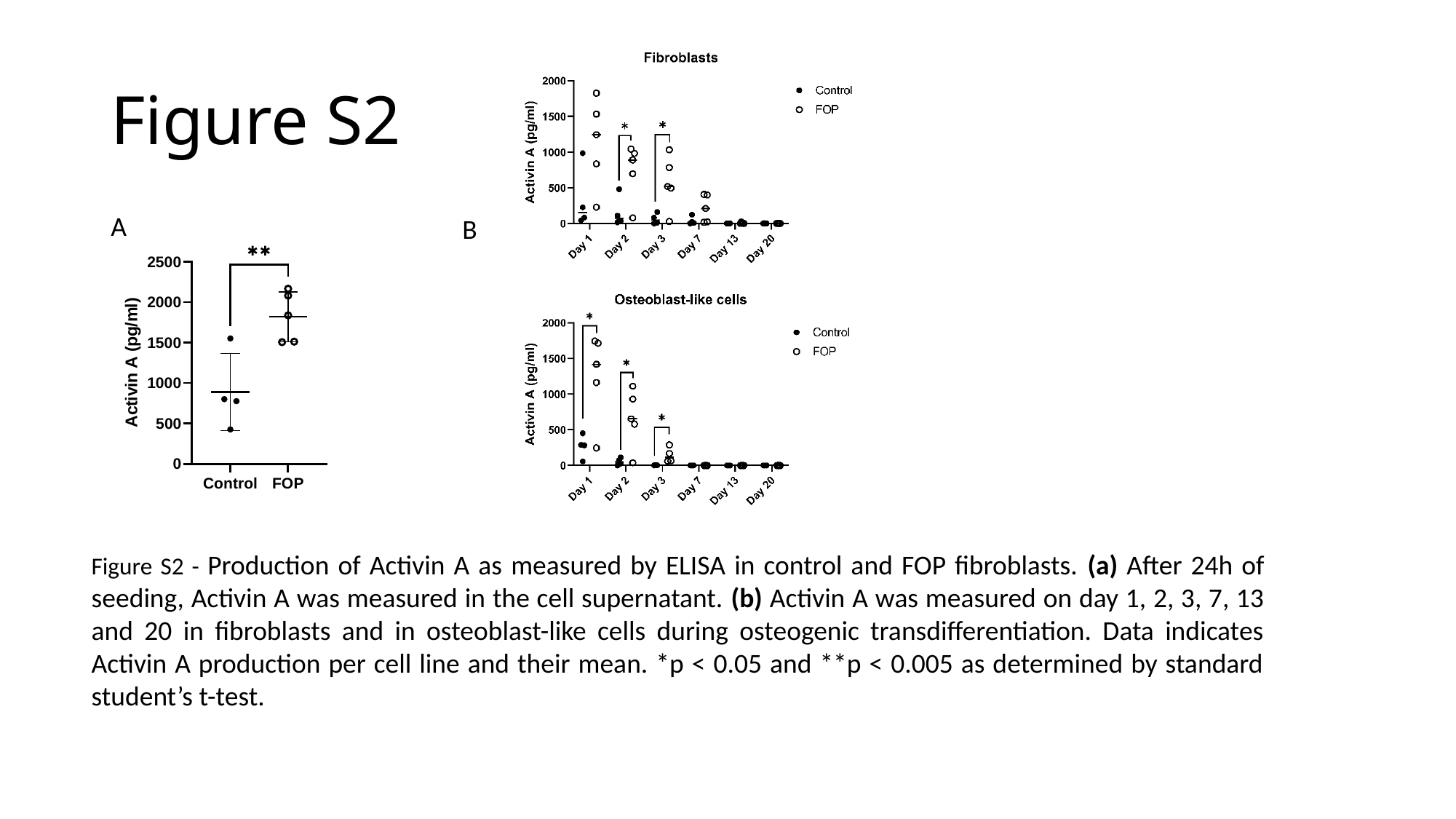

# Figure S2
A
B
Figure S2 - Production of Activin A as measured by ELISA in control and FOP fibroblasts. (a) After 24h of seeding, Activin A was measured in the cell supernatant. (b) Activin A was measured on day 1, 2, 3, 7, 13 and 20 in fibroblasts and in osteoblast-like cells during osteogenic transdifferentiation. Data indicates Activin A production per cell line and their mean. *p < 0.05 and **p < 0.005 as determined by standard student’s t-test.

## Slide 4
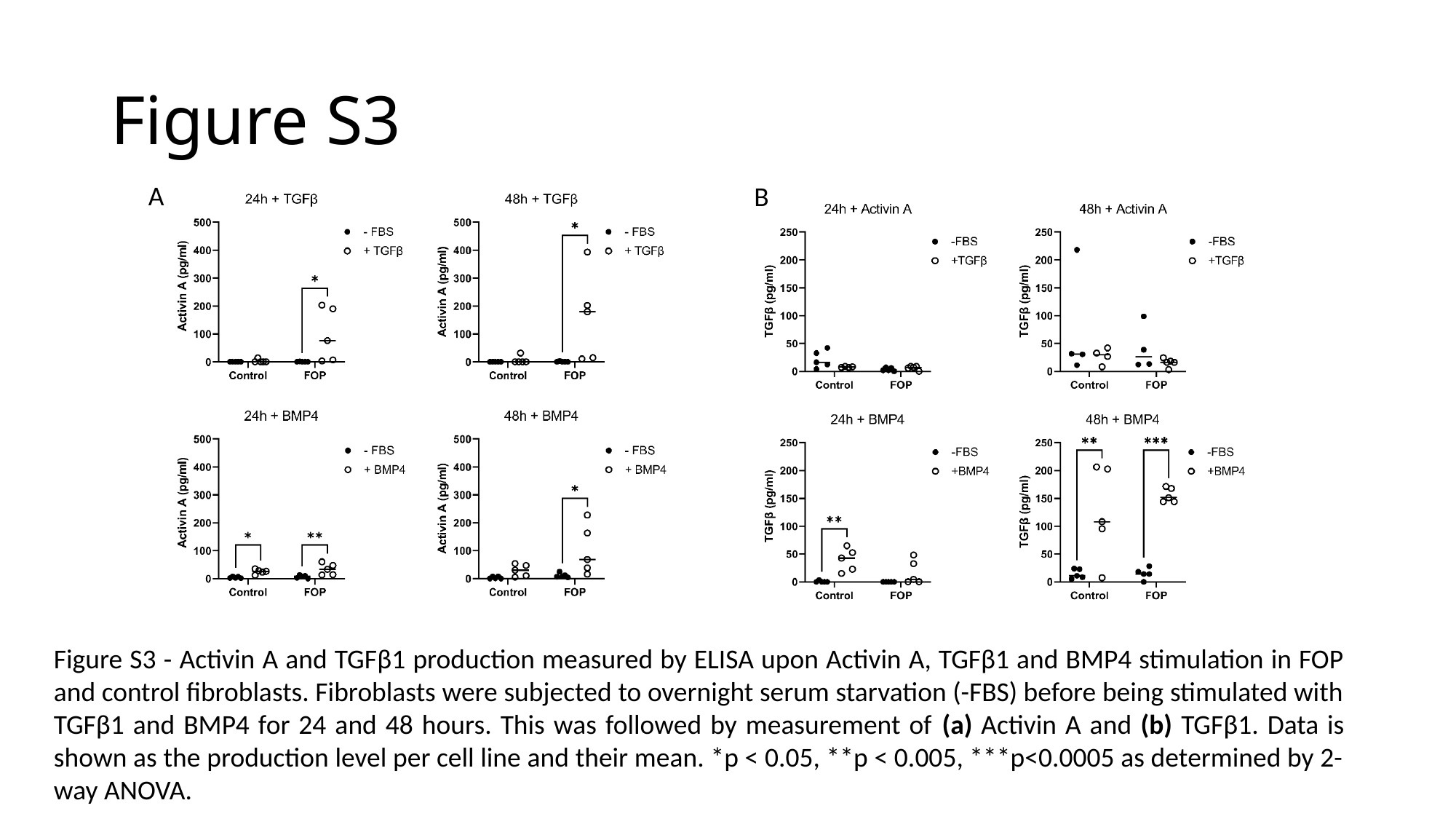

# Figure S3
A
B
Figure S3 - Activin A and TGFβ1 production measured by ELISA upon Activin A, TGFβ1 and BMP4 stimulation in FOP and control fibroblasts. Fibroblasts were subjected to overnight serum starvation (-FBS) before being stimulated with TGFβ1 and BMP4 for 24 and 48 hours. This was followed by measurement of (a) Activin A and (b) TGFβ1. Data is shown as the production level per cell line and their mean. *p < 0.05, **p < 0.005, ***p<0.0005 as determined by 2-way ANOVA.

## Slide 5
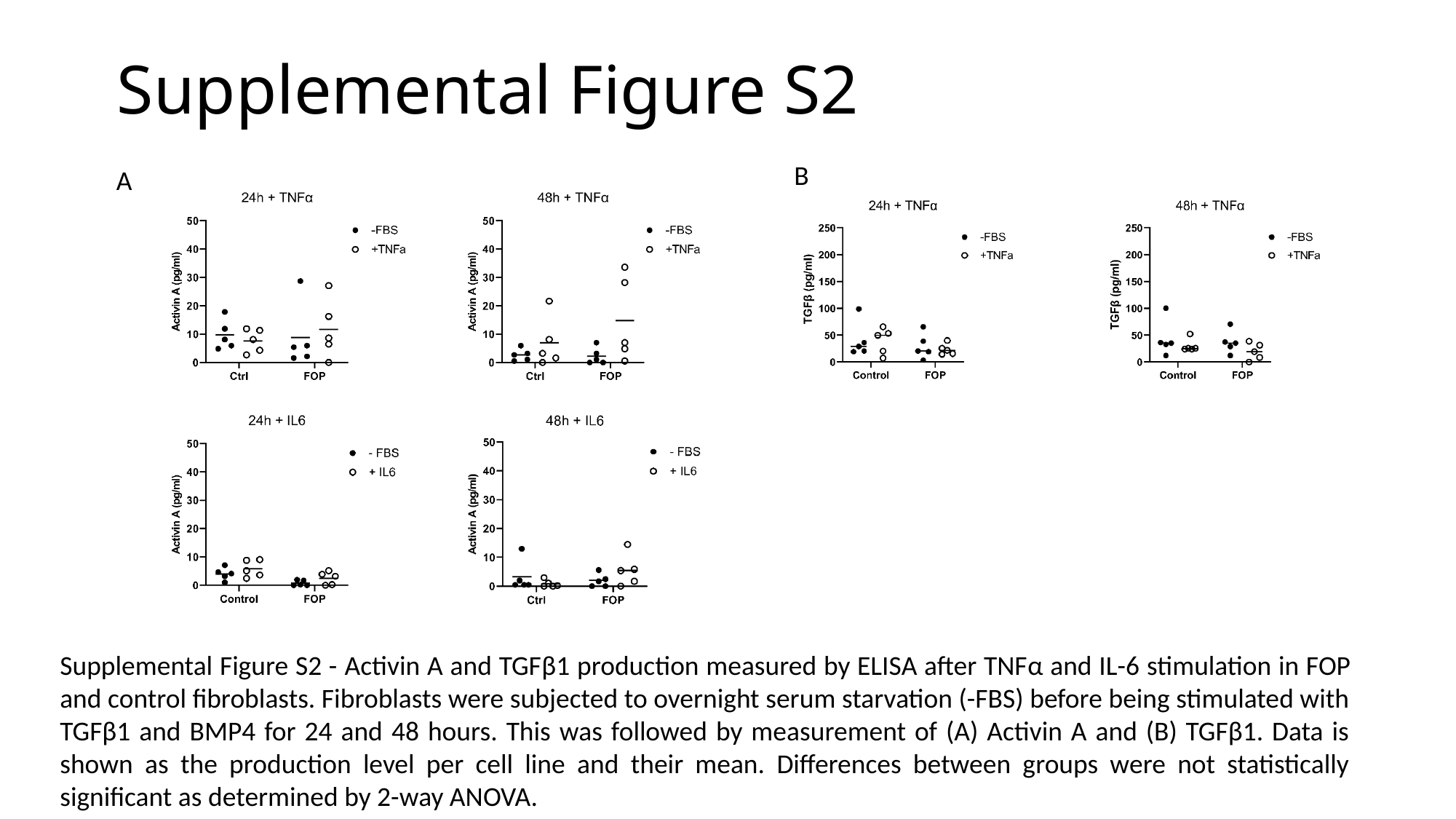

# Supplemental Figure S2
B
A
Supplemental Figure S2 - Activin A and TGFβ1 production measured by ELISA after TNFα and IL-6 stimulation in FOP and control fibroblasts. Fibroblasts were subjected to overnight serum starvation (-FBS) before being stimulated with TGFβ1 and BMP4 for 24 and 48 hours. This was followed by measurement of (A) Activin A and (B) TGFβ1. Data is shown as the production level per cell line and their mean. Differences between groups were not statistically significant as determined by 2-way ANOVA.

## Slide 6
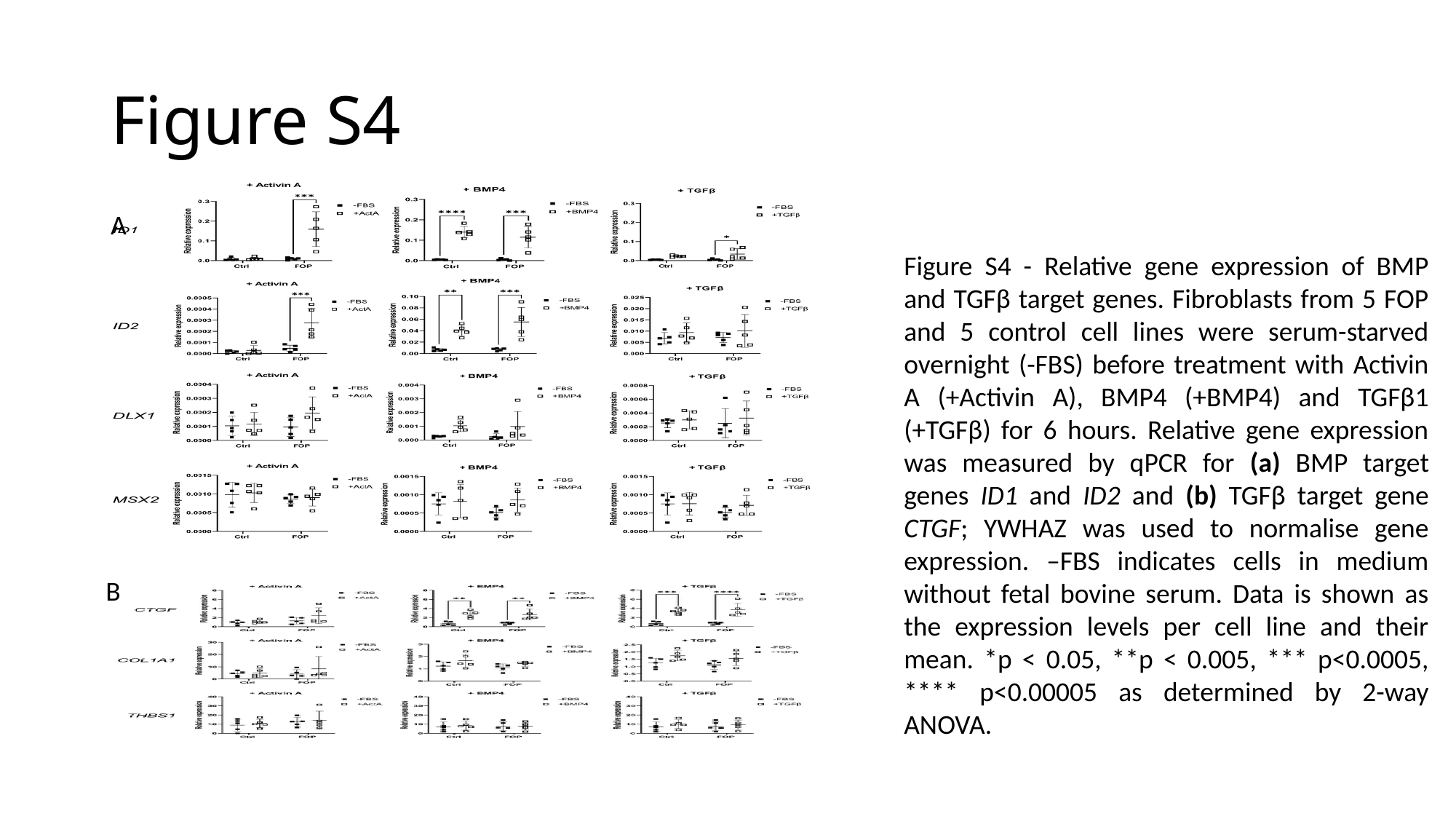

# Figure S4
A
Figure S4 - Relative gene expression of BMP and TGFβ target genes. Fibroblasts from 5 FOP and 5 control cell lines were serum-starved overnight (-FBS) before treatment with Activin A (+Activin A), BMP4 (+BMP4) and TGFβ1 (+TGFβ) for 6 hours. Relative gene expression was measured by qPCR for (a) BMP target genes ID1 and ID2 and (b) TGFβ target gene CTGF; YWHAZ was used to normalise gene expression. –FBS indicates cells in medium without fetal bovine serum. Data is shown as the expression levels per cell line and their mean. *p < 0.05, **p < 0.005, *** p<0.0005, **** p<0.00005 as determined by 2-way ANOVA.
B

## Slide 7
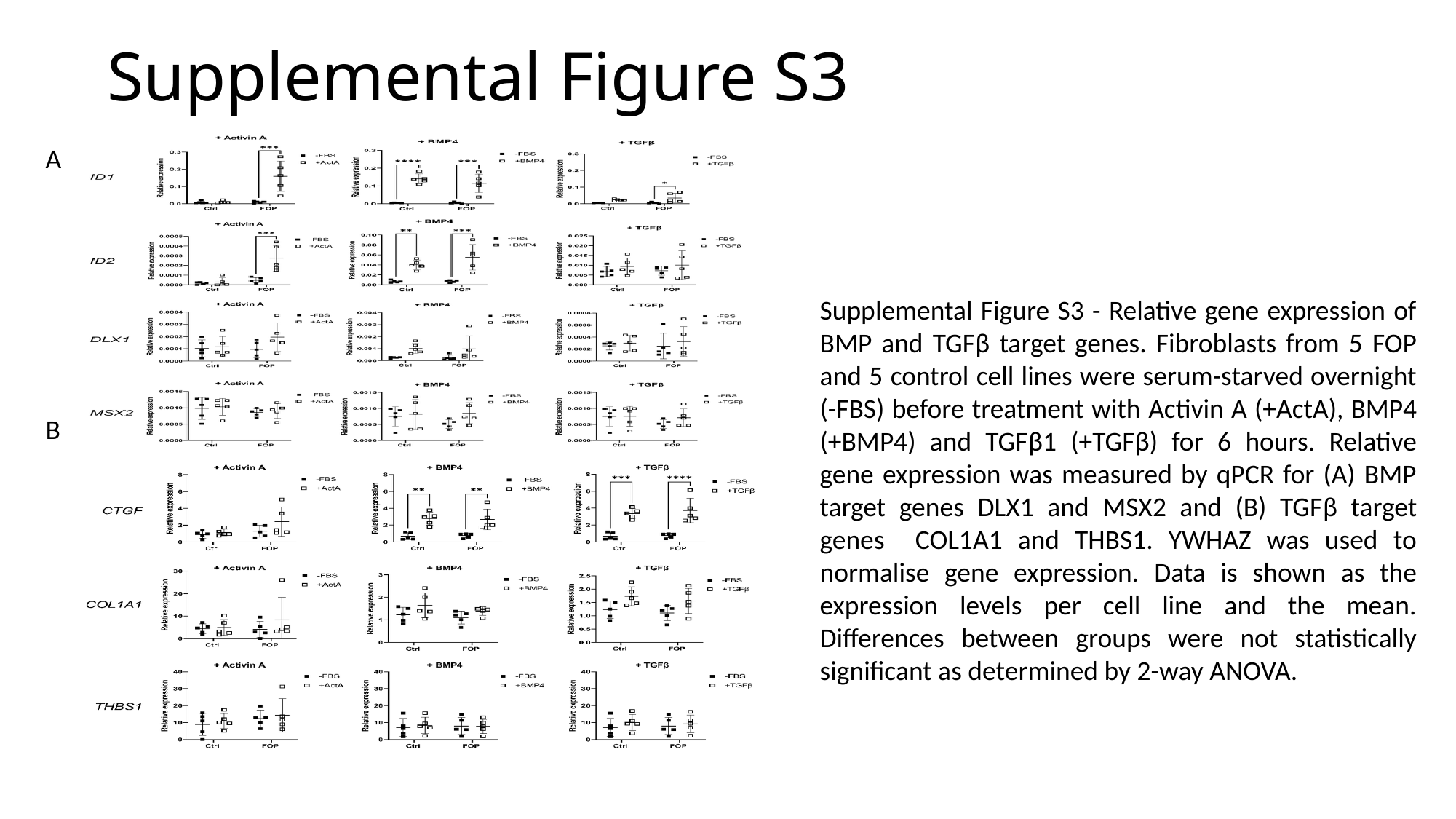

# Supplemental Figure S3
A
Supplemental Figure S3 - Relative gene expression of BMP and TGFβ target genes. Fibroblasts from 5 FOP and 5 control cell lines were serum-starved overnight (-FBS) before treatment with Activin A (+ActA), BMP4 (+BMP4) and TGFβ1 (+TGFβ) for 6 hours. Relative gene expression was measured by qPCR for (A) BMP target genes DLX1 and MSX2 and (B) TGFβ target genes COL1A1 and THBS1. YWHAZ was used to normalise gene expression. Data is shown as the expression levels per cell line and the mean. Differences between groups were not statistically significant as determined by 2-way ANOVA.
B

## Slide 8
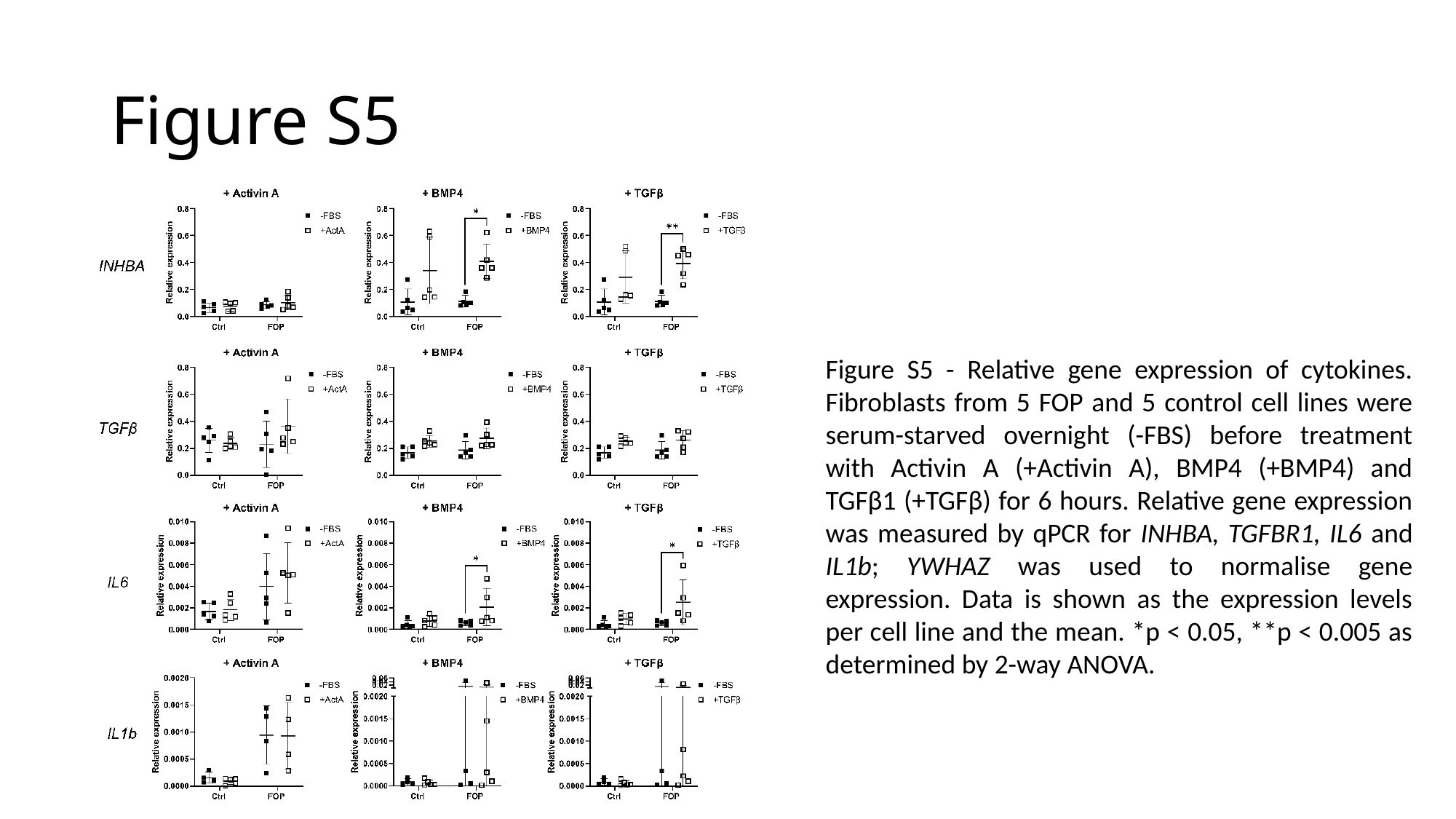

# Figure S5
Figure S5 - Relative gene expression of cytokines. Fibroblasts from 5 FOP and 5 control cell lines were serum-starved overnight (-FBS) before treatment with Activin A (+Activin A), BMP4 (+BMP4) and TGFβ1 (+TGFβ) for 6 hours. Relative gene expression was measured by qPCR for INHBA, TGFBR1, IL6 and IL1b; YWHAZ was used to normalise gene expression. Data is shown as the expression levels per cell line and the mean. *p < 0.05, **p < 0.005 as determined by 2-way ANOVA.

## Slide 9
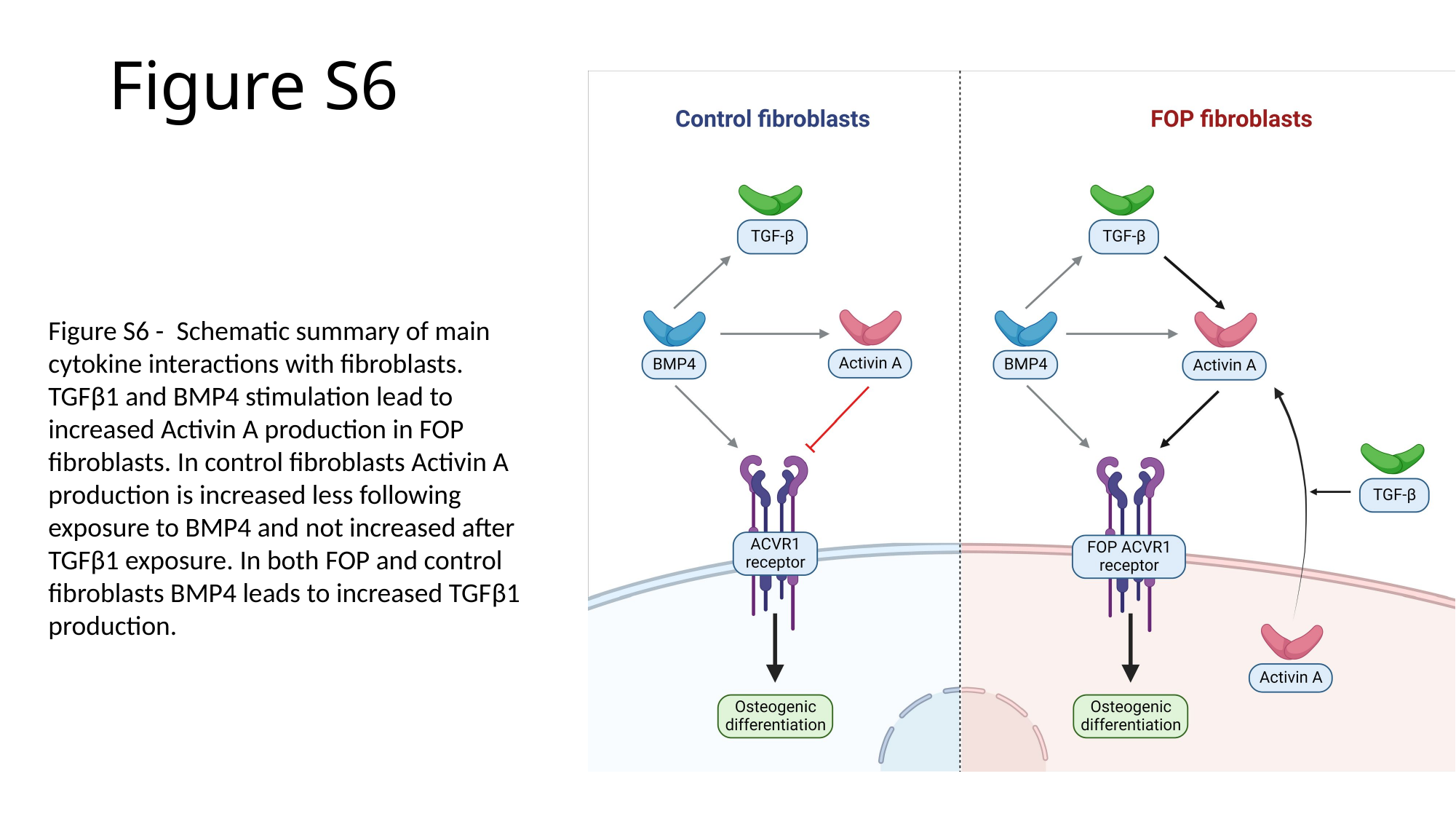

# Figure S6
Figure S6 - Schematic summary of main cytokine interactions with fibroblasts. TGFβ1 and BMP4 stimulation lead to increased Activin A production in FOP fibroblasts. In control fibroblasts Activin A production is increased less following exposure to BMP4 and not increased after TGFβ1 exposure. In both FOP and control fibroblasts BMP4 leads to increased TGFβ1 production.
