# Supplementary material for: TGF-Beta Induces Activin A Production in Dermal Fibroblasts Derived from Patients with Fibrodysplasia Ossificans Progressiva
Source: Int J Mol Sci. 2023 Jan 24;24(3):2299. doi: 10.3390/ijms24032299 (PMC9916423; doi:10.3390/ijms24032299)
Supplement: Supplementary file 1 [file ijms-24-02299-s001.zip › Supplemental figures.pptx]

## Slide 1
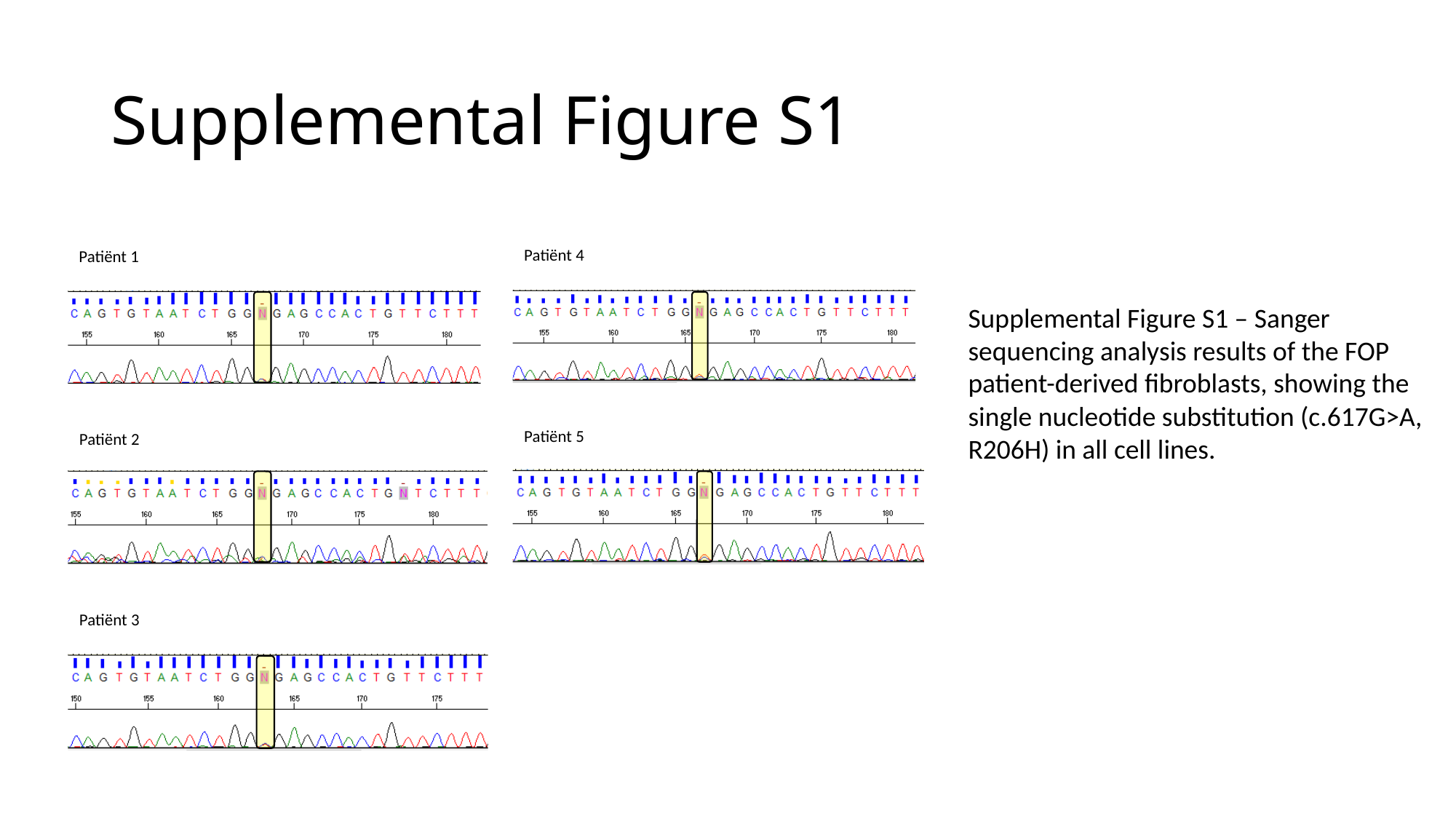

# Supplemental Figure S1
Patiënt 4
Patiënt 1
Supplemental Figure S1 – Sanger sequencing analysis results of the FOP patient-derived fibroblasts, showing the single nucleotide substitution (c.617G>A, R206H) in all cell lines.
Patiënt 5
Patiënt 2
Patiënt 3

## Slide 2
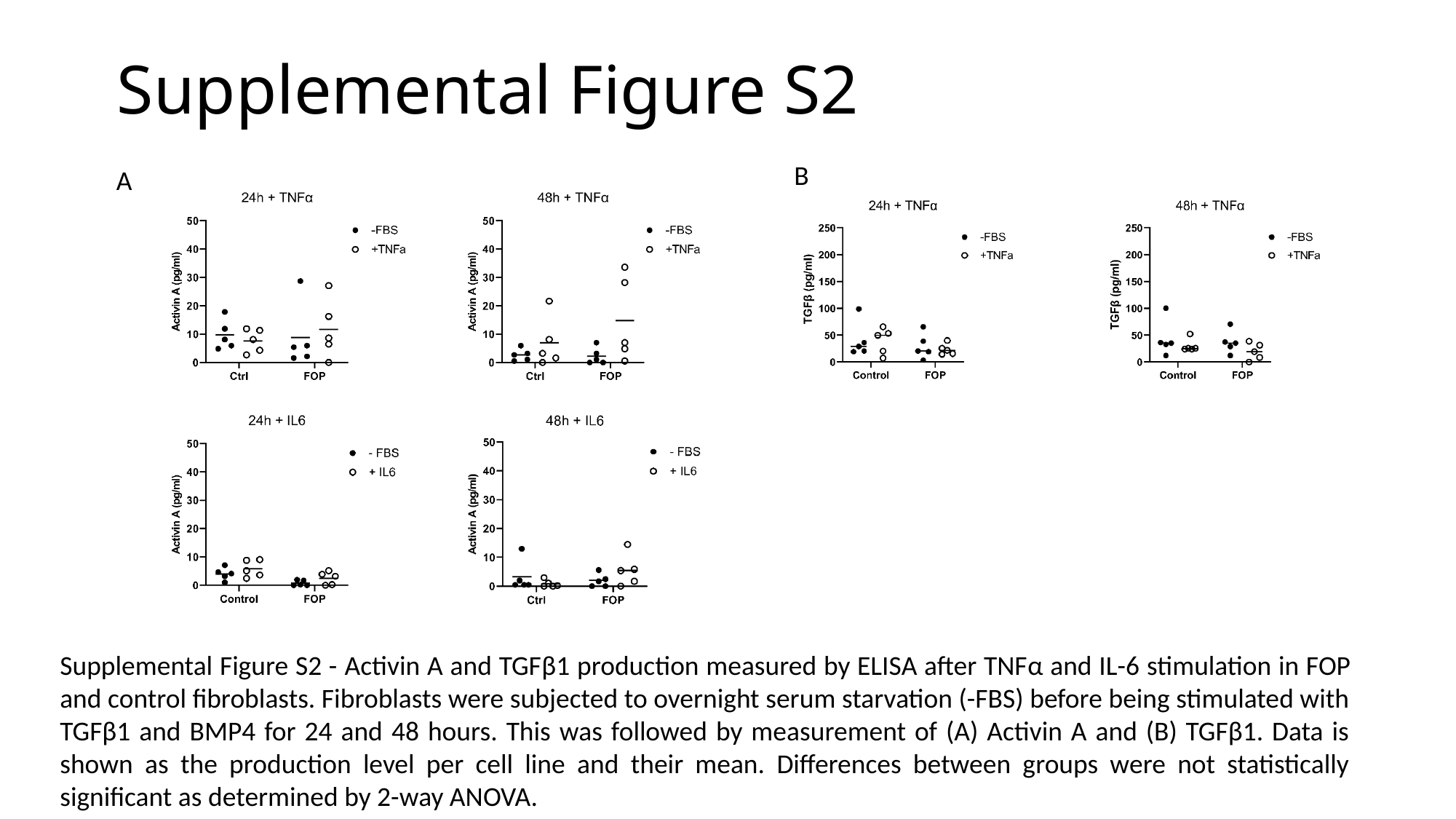

# Supplemental Figure S2
B
A
Supplemental Figure S2 - Activin A and TGFβ1 production measured by ELISA after TNFα and IL-6 stimulation in FOP and control fibroblasts. Fibroblasts were subjected to overnight serum starvation (-FBS) before being stimulated with TGFβ1 and BMP4 for 24 and 48 hours. This was followed by measurement of (A) Activin A and (B) TGFβ1. Data is shown as the production level per cell line and their mean. Differences between groups were not statistically significant as determined by 2-way ANOVA.

## Slide 3
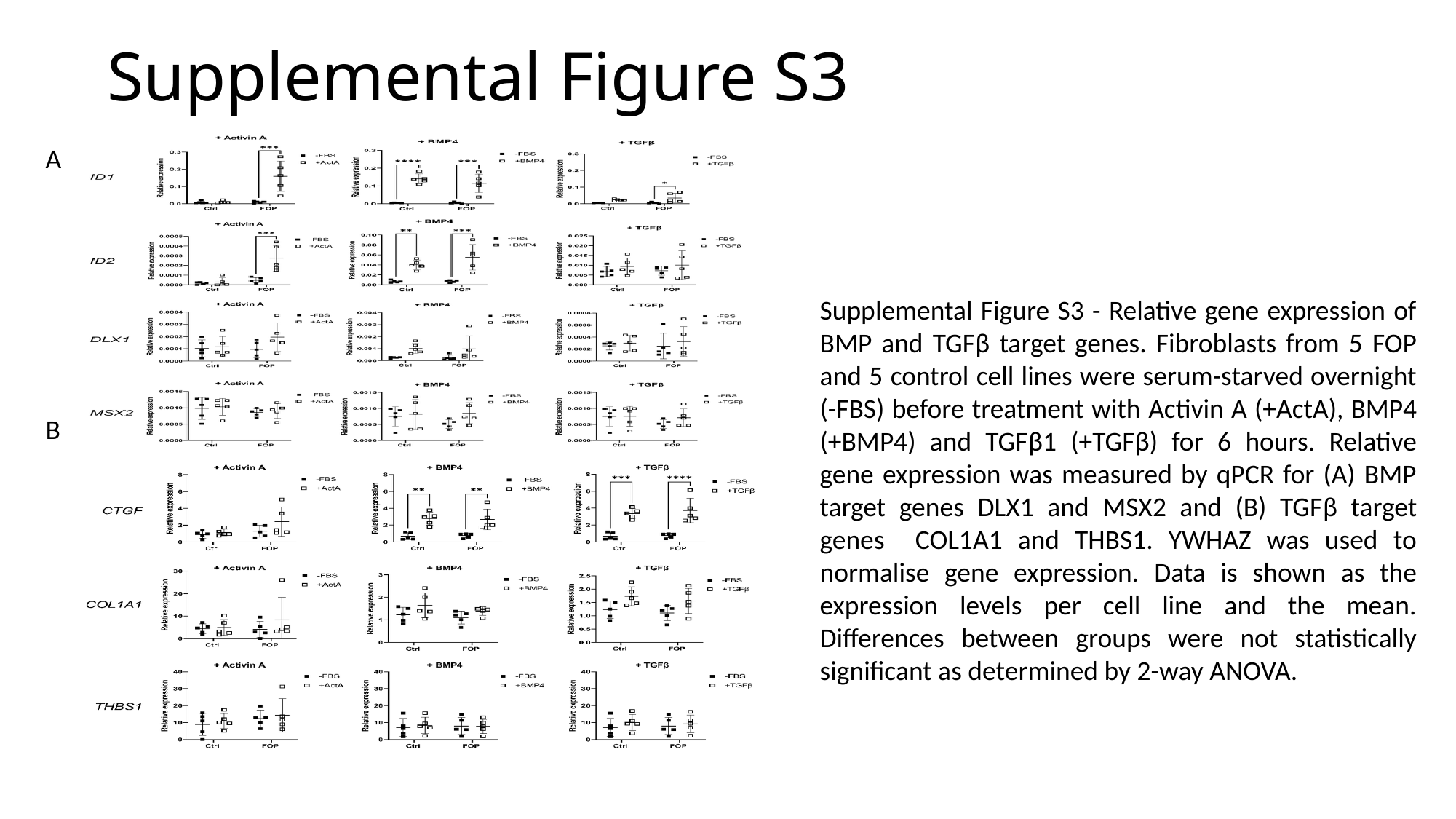

# Supplemental Figure S3
A
Supplemental Figure S3 - Relative gene expression of BMP and TGFβ target genes. Fibroblasts from 5 FOP and 5 control cell lines were serum-starved overnight (-FBS) before treatment with Activin A (+ActA), BMP4 (+BMP4) and TGFβ1 (+TGFβ) for 6 hours. Relative gene expression was measured by qPCR for (A) BMP target genes DLX1 and MSX2 and (B) TGFβ target genes COL1A1 and THBS1. YWHAZ was used to normalise gene expression. Data is shown as the expression levels per cell line and the mean. Differences between groups were not statistically significant as determined by 2-way ANOVA.
B
