# Supplementary figures and images for: TGF-Beta Induces Activin A Production in Dermal Fibroblasts Derived from Patients with Fibrodysplasia Ossificans Progressiva
Source: Int J Mol Sci. 2023 Jan 24;24(3):2299. doi: 10.3390/ijms24032299 (PMC9916423; doi:10.3390/ijms24032299)

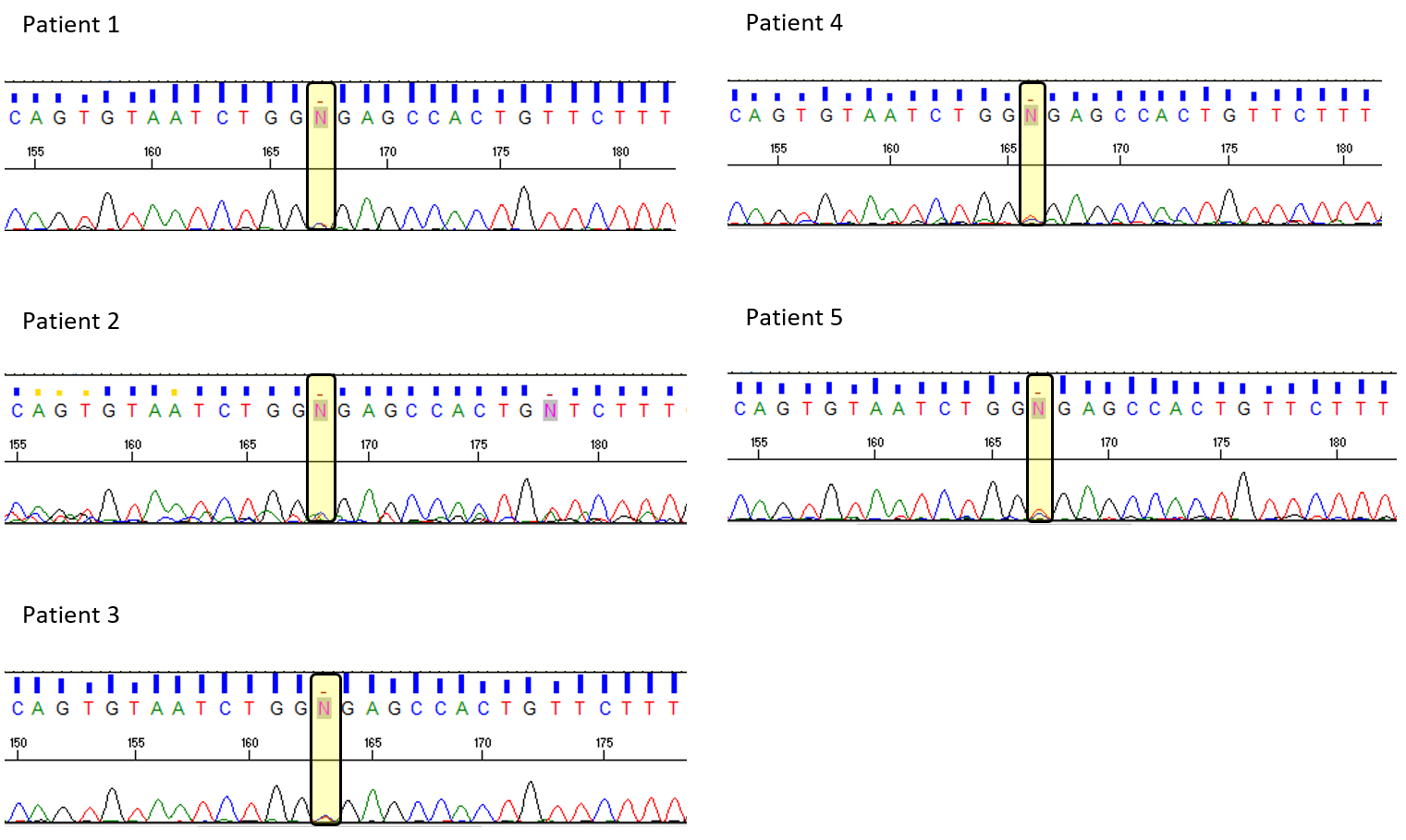

Supplement: Supplementary file 1 [file ijms-24-02299-s001.zip › Supplemental figure 1.tif]

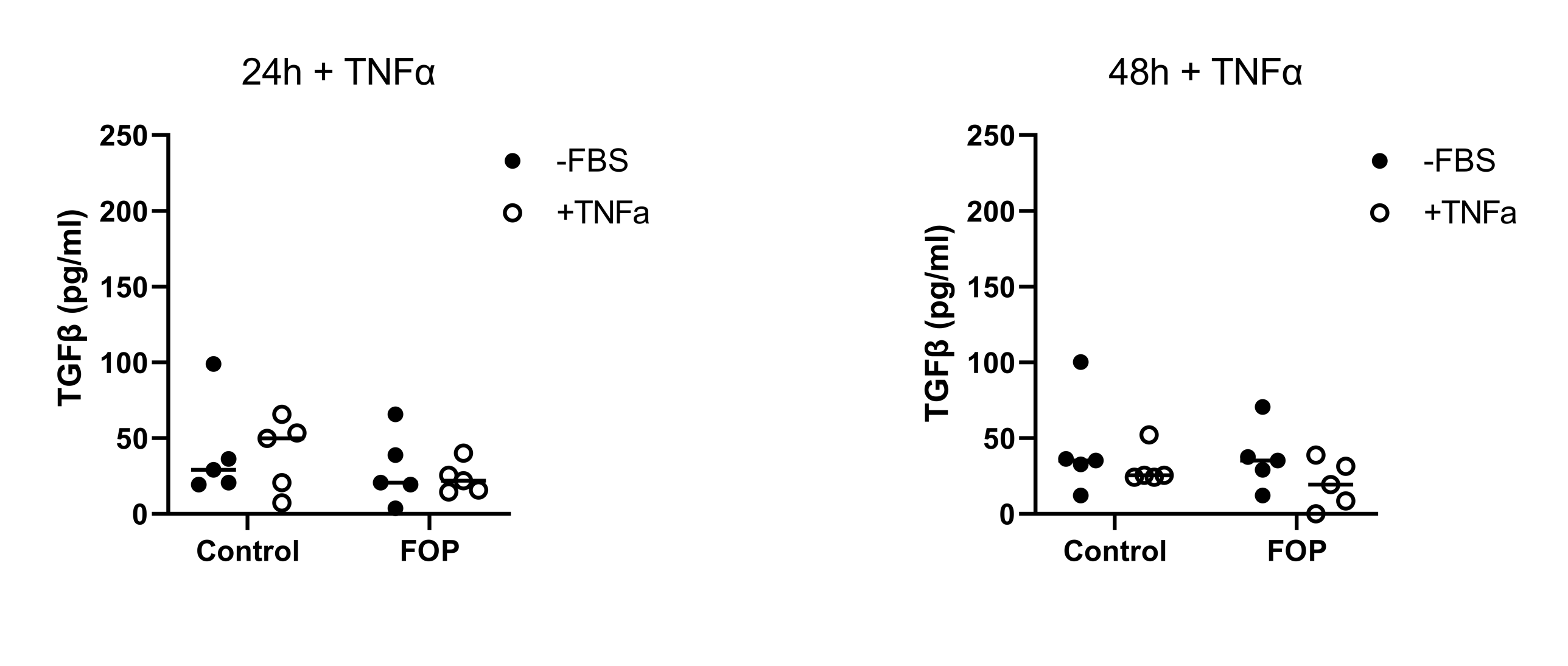

Supplement: Supplementary file 1 [file ijms-24-02299-s001.zip › Supplemental figure 2B.tif]

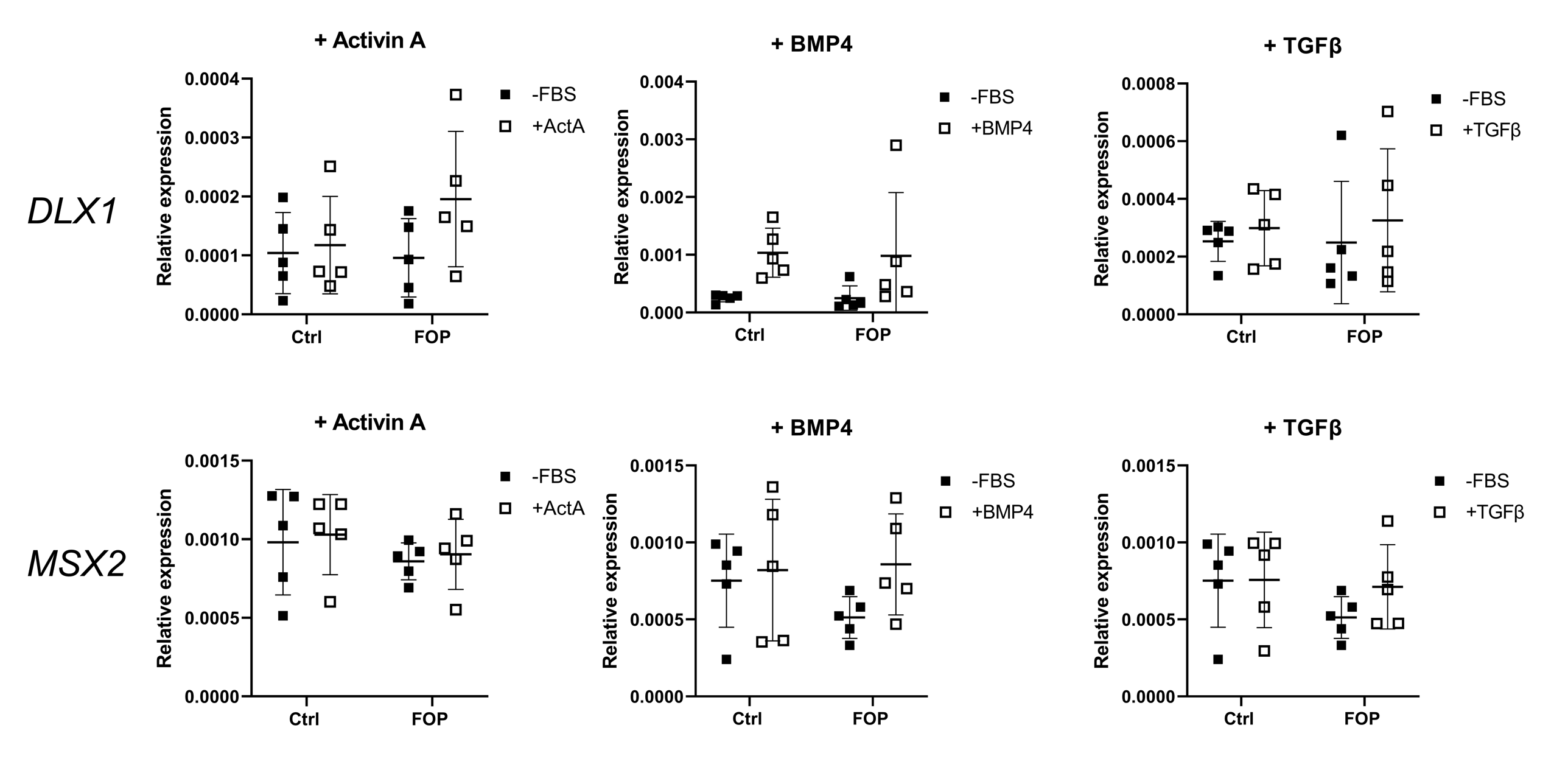

Supplement: Supplementary file 1 [file ijms-24-02299-s001.zip › Supplemental figure 3A.tiff]

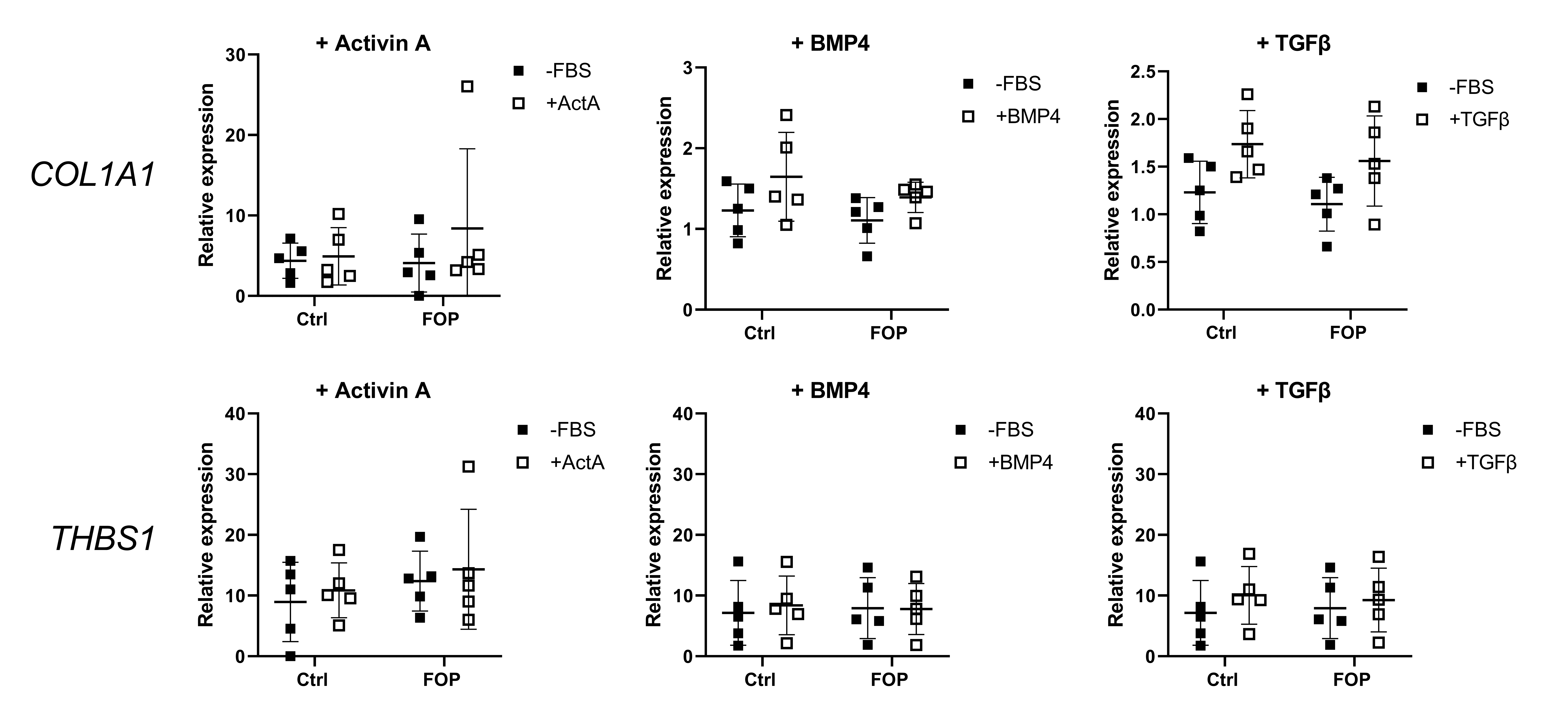

Supplement: Supplementary file 1 [file ijms-24-02299-s001.zip › Supplemental figure 3B.tiff]
